# Pseudogout of the temporomandibular joint: a case report with systematic literature review

**DOI:** 10.22514/jofph.2025.004

**Published:** 2025-03-12

**Authors:** Matteo Val, Mirko Ragazzo, Anna Colonna, Marco Ferrari, Edoardo Ferrari Cagidiaco, Daniele Manfredini, Luca Guarda Nardini

**Affiliations:** ^1^Department of Biomedical Technologies, School of Dental Medicine, University of Siena, 53100 Siena, Italy; ^2^Unit of Oral and Maxillofacial Surgery, Ca’Foncello Hospital, 31100 Treviso, Italy

**Keywords:** Calcium pyrophosphate dihydrate deposition disease, Pseudogout, Temporomandibular joint, Systematic review, Epidemiology

## Abstract

**Background**: Calcium pyrophosphate dihydrate deposition disease (CPPD) is a metabolic disease resulting in acute arthritis. CPPD often affects joints containing fibrocartilage. The purpose of this review is to examine the clinical presentations, prevalence, and treatment modalities associated with CPPD when it affects the temporomandibular joint (TMJ). **Methods**: A search, following PRISMA guideline, was conducted in various electronic databases (PubMed, Scopus, Web of Science) to find relevant studies about CPPD in the temporomandibular joint. The search spanned from 01 January 1980, to 31 January 2024. A case report was also presented. **Results**: A systematic review of literature identified 64 papers, reaching a total of 74 cases of CPPD of the temporomandibular joint TMJ. CPPD is a condition that typically affects middle-aged or older patients, with an average age of around 60 at the time of diagnosis. Females are affected more frequently than males. Most cases involve unilateral TMJ involvement, and common symptoms include pain, reduced mouth opening, and swelling. Different papers also describe severe stages of the invasion of muscles, parotid gland, and even brain structure. Surgery has the preferred treatment option for most Authors and is mandatory for late-stage lesions. The recurrence rate is extremely low. **Conclusions**: CPPD is an uncommon, locally invasive, and typically benign condition that rarely affects the TMJ. Distinguishing CPDD in the TMJ from other neoplasms poses diagnostic challenges. A definitive diagnosis necessitates histological examination and quantitative microanalysis. In our patient, successful excision of CPDD in the TMJ was achieved using an external approach. **The PROSPERO Registration**: PROSPERO number is CRD42024558402.

## 1. Introduction

Calcium pyrophosphate dihydrate deposition disease (CPPD) is a metabolic disorder that causes non-infectious inflammation in joints and can lead to calcification either within or around the joint [1]. In 1962, Kohn *et al.* [2] were the first to document the presence of CPPD crystals in the synovial fluid of the knee in patients with “chondrocalcinosis”, characterized by cartilage calcifications on standard radiographs [3] along with acute symptoms associated with gout. Chondrocalcinosis is commonly used as a marker for the presence of CPPD in population-based studies, and its occurrence is closely linked to aging. Radiological evidence of chondrocalcinosis is observed in 6% to 15% of patients above the age of 60. This prevalence increases to 30% to 40% in patients aged over 80 years [1]. Despite the knowledge on the disease, many instances are probably not being diagnosed correctly [4].

Furthermore, Willekens *et al*. [5] pointed out that individuals with peripheral calcific disease are more likely to have involvement of the temporomandibular joint (TMJ) than previously believed.

The involvement of the TMJ by CPPD exhibits signs and symptoms that closely resemble various other temporomandibular joint disorders. Consequently, inexperienced professionals may attempt multiple management approaches, often resulting in a delayed or missed diagnosis [6, 7], which is a common occurrence in this type of pathology [8, 9, 10, 11]. Therefore, accurate classification of the patient using evidence-based methods is imperative [12, 13, 14].

Information on CPPD is currently limited. The prevalence of this condition remains unknown, and milder forms may be underdiagnosed. Additionally, effective treatment for CPPD affecting the temporomandibular joints has yet to be established, unlike for other joint involvements.

Based on this premise, it seems interesting to get deeper into the description of pseudogout of the TMJ. In this manuscript, we report a clinical case of large TMJ pseudogout as well as a systematic review of the existing literature, with the aim to share information about the prevalence, management and follow-up of patients with CPPD.

## 2. Case presentation

An 87-year-old Caucasian female was referred to the Unit of Oral and Maxillofacial Surgery in September 2022 for the evaluation of a firm swelling located over her left TMJ. The patient had been experiencing chronic left TMJ pain for over 2 years. Despite seeking medical advice from multiple healthcare providers during this period, she did not receive a definitive diagnosis. Her dentist tried different medical treatment, including anti-inflammatory (ibuprofen) associated with amoxicillin + clavulanic acid 875 + 125 mg (1 pill every 8 hours for 7 days), without success. Then a second dentist produced an oral device trying to reduce overload and the swelling to the TMJ, but there were no improvements. Her past medical history includes diabetes, hypercholesterolemia and hypoacusia. She was receiving treatment that included aspirin, insulin, metformin, gliclazide and statins. The patient denied any history of trauma or infection to the jaw area.

Upon examination, a localized swelling was identified in the left pre-auricular region, measuring approximately 30 mm. The area exhibited tenderness upon palpation and showed a firm, bony texture. The patient complained of mouth limitation and lateral deviation of the mandible when mouth opening. No other symptoms were reported by the patient.

A computerized tomography (CT) was performed due to the suspect of a neoformation of the left TMJ. From the radiological examination was highlighted a massive radiopaque lesion (major diameter 39 mm) was shown that embroidered the head of the left condyle, which appeared degenerated as the glenoid fossa, with some minor defects also evident in the roof of the fossa (Fig. 1). Based on the patient’s primary clinical characteristics and radiological findings, the differential diagnosis included tumor or tumor-like conditions, such as pigmented villonodular synovitis (PVNS), synovial chondromatosis, and the potential for malignant chondrosarcoma of the right temporomandibular joint (TMJ). The patient underwent surgical removal of the mass under general anaesthesia through a preauricular approach followed by tissue dissection. During the procedure, the mass was found to be an encapsulated soft tissue mass originating and surrounding from the circumference of the articular disk (Fig. 2). Once the capsule was cute a nodular, friable, gritty granulomatous tissue emerged. It was excised in two separate portions. Arthroplasty was performed using a burr to re-establish the correct shape of the mandibular condyle. The incision is subsequently closed using layered sutures. Upon microscopic examination, it was observed that there were nodules of birefringent crystalline material and calcifications present, which were associated with reactive chondroblasts and histiocytes. The integrity of the crystals during processing indicated the presence of calcium pyrophosphate crystal deposition, known as pseudogout, in the TMJ. The patient had a smooth recovery with no facial weakness and reported an increase in maximal inter-incisal opening from 19 mm to 36 mm during the one-year follow-up examination after surgery. Leftward deviation of the mandible only occurred at maximum opening, and there were no changes in occlusion reported. Pain and swelling had completely resolved.

**Fig. 1.** Radiographic evaluation pre and post-surgical excision. (A) illustrates the coronal view of a computed tomography (CT) scan prior to surgical intervention, while (B) represents the axial view of the CT scan before surgery. Conversely, (C) depicts the radiological findings following the surgical excision of the lesion in a coronal view, and (D) presents the axial view of the CT scan.

**Fig. 2.** Surgical excision. (A) shows the clinical appearance following surgical removal of superficial tissue over the temporomandibular joint (TMJ), displaying nodular, friable, gritty granulomatous tissue. (B) represents the surgical specimens, and (C) shows the TMJ with the mass completely removed.

## 3. Materials and methods

### 3.1 Electronic database search

To find relevant studies about calcium pyrophosphate dihydrate crystal deposition disease in the temporomandibular joint, a search was conducted in the following electronic databases PubMed, Web of Science and Scopus following the PRISMA guidelines [15]. For the PRISMA checklist, please see **Supplementary material**. The search terms used were “Calcium pyrophosphate dihydrate crystal deposition disease temporomandibular joint”, “CPPD temporomandibular joint” and “Pseudogout temporomandibular joint”. Keywords and Medical Subject Headings (MeSH) terms were used to combine the search on the aforementioned databases. In addition, the “Related articles” option on the PubMed homepage was considered, and a manual search of article references was conducted to capture any additional relevant reports. The search spanned from 01 January 1980, to 31 January 2024. The review has been registered in PROSPERO with the Identification Number (ID) number CRD42024558402. The full list of keywords used are:

- (“chondrocalcinosis” [MeSH Terms] OR “chondrocalcinosis” [All Fields] OR “pseudogout” [All Fields]) AND (“temporomandibular joint” [MeSH Terms] OR (“temporomandibular” [All Fields] AND “joint” [All Fields]) OR “temporomandibular joint” [All Fields])

- (“chondrocalcinosis” [MeSH Terms] OR “chondrocalcinosis” [All Fields] OR (“calcium” [All Fields] AND “pyrophosphate” [All Fields] AND “dihydrate” [All Fields] AND “crystal” [All Fields] AND “deposition” [All Fields] AND “disease” [All Fields]) OR “calcium pyrophosphate dihydrate crystal deposition disease” [All Fields]) AND (“temporomandibular joint” [MeSH Terms] OR (“temporomandibular” [All Fields] AND “joint” [All Fields]) OR “temporomandibular joint” [All Fields])

### 3.2 Inclusion and exclusion criteria

For the review, only the studies that met the following criteria were included: (i) they were original case series or case reports, (ii) they documented Pseudogout, and (iii) they involved the temporomandibular joint in patients of any age. Articles that are not specific to the topic or are published in languages other than English were excluded; in particular, it was necessary to prevent misinterpretation by authors whose second language is English. Reports involving tumor calcinosis or with insufficient data.

### 3.3 Data extraction

Two of the authors (MV; AC) thoroughly reviewed literature data and carefully selected all the studies that met the eligibility criteria. All relevant data, such as demographics, clinical characteristics, outcomes and follow-up, were extracted and recorded from each study. Any discrepancies were resolved through discussion with the senior authors (DM, LGN).

### 3.4 Descriptive analysis

The initial plan was to conduct a meta-analysis for this systematic review. However, since the studies showed a relevant degree of heterogeneity, it was not feasible to proceed with this approach. As a result, a descriptive analysis of the studies was conducted instead.

### 3.5 Outcome

The main results analyzed in the studies were:

Provide an estimate of the real prevalence of CPPD;

Investigate the co-occurrence of medical conditions and underlying systemic factors that contribute to the development of CPPD;

Describe the most common signs and symptoms related to CPPD;

Identify the most effective treatment option.

Secondary Outcomes were:

Evaluate the presence of relapse;

Highlight the best radiological methods to study this pathology.

### 3.6 Risk of bias evaluation

Two authors (MV/MR) evaluated these aspects for each study: incomplete data evaluation, evaluation report, and other sources of biased selective results. Each research study underwent classification as either low risk, high risk or undefined risk. Any discrepancies in the assessment process were resolved through deliberation or mediation involving a third researcher (DM).

Regrettably, the presence of solely case reports and case series, along with significant disparities in treatments administered even within the same patient series, precluded a comprehensive assessment of bias.

## 4. Results

A total of 125 papers were found. After the screening procedures for inclusion in the review, 64 case report/case series were identified. The flowchart depicting the article selection process for all search queries is available in Fig. 3.

**Fig. 3.** **Flowchart illustrating the procedure for article selection.** Legend: CPPD: Calcium pyrophosphate dihydrate deposition disease; TMJ: temporomandibular joint.

### 4.1 Population’s characteristics

Table 1 (Ref. [8, 9, 10, 16, 17, 18, 19, 20, 21, 22, 23, 24, 25, 26, 27, 28, 29, 30, 31, 32, 33, 34, 35, 36, 37, 38, 39, 40, 41, 42, 43, 44, 45, 46, 47, 48, 49, 50, 51, 52, 53, 54, 55, 56, 57, 58, 59, 60, 61, 62, 63, 64, 65, 66, 67, 68, 69, 70, 71, 72, 73, 74, 75, 76, 77, 78, 79, 80, 81, 82, 83, 84, 85, 86, 87]) presents the characteristics of the studies included, which were published between 1982 and 2023. These studies encompassed a total of 64 papers, involving 74 patients. Females outnumbered males (F:M = 46:28). The mean age at the moment of diagnosis ranged between 60.95 ± 13.02. Almost all patients (70 out of 74) had a unilateral TMJ involvement. Only 22 had a recorded medical history concerning any possible comorbidities. The three most frequent comorbidities were hypertension, hypercholesterolemia and diabetes. Additionally, there were four cases of patients suffering from gout.

**Table 1.** Characteristics of the patients.

Study first Author	Year	N of patients	Sex	Age	Localization	History of the lesion	Systemic diseases
Good *et al*. [20]	1982	1	M	59	Right TMJ	Acute origin	Not reported
Zemplenyi *et al*. [29]	1985	2	F	51	Left TMJ	No description	Not reported
Gross *et al*. [30]	1987	3	F	59	Left TMJ	History of generalized arthritis affecting multiple joints, which has been managed with localized steroid injections.	Not reported
Hutton *et al*. [22]	1987	4	F	78	Right TMJ	Acute origin	Not reported
		5	F	76	Right TMJ	Acute origin	Not reported
		6	F	68	Right TMJ	Acute origin	Polymyalgia rheumatica, for which she had been treated with prednisolone
Kamatani *et al*. [43]	1987	7	M	57	Left TMJ	Trauma 6 year before with mild symptoms	Diabetes mellitus
Mogi *et al*. [61]	1987	8	F	54	Right TMJ	8 years history of pain and swelling since thel esion was highlighted	Not reported
Lambert *et al*. [32]	1990	9	M	41	Left TMJ	18 years since the lesion was highlighted	Not reported
Dijkgraaf *et al*. [44]	1992	10	F	53	Left TMJ	8 months before had a loud click in her TMJ with pain and disk displacement at the MRI	Not reported
Magno *et al*. [33]	1992	11	F	53	Left TMJ	The patient was involved in a motor vehicle accident many years ago, during which she suffered a cervical vertebral fracture	Not reported
Chuong *et al*. [46]	1995	12	F	65	Bilateral TMJ	The patient has an 8-year history of bilateral temporomandibular joint (TMJ) pain, periodic popping and locking, and frequent restricted mouth opening, particularly during episodes of frank preauricular swelling	Hypothyroidism, hypertension, hiatal hernia, and von Willebrand’s disease.
Pynn *et al*. [45]	1995	13	M	58	Left TMJ	The patient presented with a history of swelling persisting for 18 months. The swelling manifested abruptly and maintained its size until one month prior to the referral, when it significantly increased before gradually returning to its original size following a two-week course of oral acetylsalicylic acid	Not reported
Allias-Montmayeur *et al*. [23]	1997	14	M	52	Left TMJ	Patient presents with a limited mouth opening, mandibular deviation, and pre-auricular swelling persisting for 4 years	Not reported
		15	F	46	Left TMJ	Acute attack of pain in the left TMJ. Same episode 3 years before. Pre-auricular swelling	Not reported
Kurihara *et al*. [51]	1997	16	M	85	Right TMJ	Swelling in the right TMJ region, which he had suffered for 2 years. Pain during mastication	Not reported
Onodera *et al*. [69]	1997	17	F	48	Left TMJ	Swelling for 3 years, restricted mouth opening	Not reported
Vargas *et al*. [70]	1997	18	F	66	Left TMJ	2-month history of swelling with tenderness over the left pre-auricular region with pain with restriction of jaw opening	Not reported
Jordan *et al*. [16]	1998	19	M	80	Right TMJ	The patient presented with a one-month history of sudden worsening of right-sided hearing loss following descent in an airplane flight. He denied experiencing any temporomandibular joint (TMJ) pain, trismus, jaw popping, or clicking. A right-sided middle ear effusion was observed and treated with myringotomy, but reaccumulated rapidly.	Hypertension, hypercholesterolemia, and gout
Strobl *et al*. [71]	1998	20	F	51	Left TMJ	The patient presented with an 18-month history of progressive left-sided temporomandibular joint (TMJ) pain and trismus. There was no reported history of trauma or systemic joint disease	Not reported
Goudot *et al*. [72]	1999	21	F	63	Left TMJ	10-year history of left TMJ pain and restricted mouth opening	Not reported
Grant *et al*. [28]	1999	22	F	65	Left TMJ	The patient presented with a 2- to 3-month history of progressive left-sided facial fullness, discomfort, and intermittent facial swelling. The discomfort was alleviated after a course of antibiotic medications and prednisone for suspected parotiditis, although the swelling persisted. The patient denied experiencing any paresthesias, nasal issues, otalgia, or swallowing difficulties. Her medical history revealed longstanding bilateral middle ear infections, necessitating the placement of a left-sided myringotomy tube	Not reported
Nakagawa *et al*. [73]	1999	23	F	60	Right TMJ	The patient has a 1-year history of swelling in the right preauricular region and has reported a l-month history of TMJ discomfort and trismus	Not reported
		24	F	45	Left TMJ	The patient reported a progressive increase in left temporomandibular joint (TMJ) pain, which commenced several years prior to seeking medical attention. The pain was described as a burning sensation and was exacerbated during jaw movement.	Not reported
Pynn *et al*. [50]	1995	25	M	58	Left TMJ	The patient presented with painless swelling in the left preauricular region persisting for approximately 18 months. The swelling had a sudden onset and remained unchanged in size until one month prior to referral, when it abruptly increased before slowly returning to its original size following two weeks of oral acetylsalicylic acid (aspirin) therapy	Not reported
Aoyama *et al*. [74]	2000	26	F	45	Left TMJ	The patient presented with painful swelling in the left preauricular area and reported experiencing a clicking noise when opening her mouth for over 30 years. Additionally, eight years ago, she experienced pain in the same area, which subsided following occlusal adjustment and the prescription of a muscle relaxant	Not reported
Li-Yu *et al*. [34]	2000	27	F	72	Left TMJ	Excruciating pain in her left ear. Initial treatment for suspected otitis media included antibiotics with no improvement noted. The pain gradually extended to her left cheek and jaw, with some localized swelling and tenderness over the TMJ area	Hysterectomy in 1985 and breast cancer in 1993
Eriksson *et al*. [75]	2001	28	F	72	Right TMJ	The patient has been experiencing chronic pain in the right TMJ for several years, with the pain intensifying over time and becoming more pronounced during chewing. Additionally, the patient has observed a minor swelling in the joint and has reported experiencing clicking sensations. The patient also recalls experiencing trauma to the chin during childhood	Not reported
Olin *et al*. [24]	2001	29	F	51	Left TMJ	The patient developed a pre-auricular swelling on the left side that has been persistent for two months	Not reported
Osano *et al*. [76]	2003	30	M	40	Left TMJ	The patient presented with pain and swelling in his left TMJ. He had previously encountered similar symptoms two years ago, which were alleviated with intravenous antibiotic infusion	Not reported
Marsot-Dupuch *et al*. [35]	2004	31	F	70	Right TMJ	The patient has a 10-year history of right TMJ pain and an ear lump. She was referred due to acute exacerbation, new onset of left TMJ pain, and right-sided hearing loss	Not reported
		32	M	53	Left TMJ	The patient presents with a 1-year history of acute left aural fullness and conductive hearing loss. Examination revealed otitis media, for which the patient had previously undergone myringotomy and tube placement at an external medical facility, with no improvement in symptoms. The patient denied experiencing otorrhea, otalgia, vertigo or tinnitus	Diabetes mellitus
Meul *et al*. [77]	2005	33	M	54	Left TMJ	Patient has a 3-year history of painless swelling in the left preauricular region	The individual experienced paraplegia as a result of a motor-vehicle accident 25 years ago, and also has non-insulin dependent diabetes
Smolka *et al*. [25]	2005	34	F	74	Left TMJ	Extremely painful preauricular swelling on the left side	Not reported
Nicholas *et al*. [52]	2007	35	F	35	Left TMJ	The patient presented with a 2-month history of left-sided otalgia and an external auditory canal lesion that did not respond to antibiotic treatment. Additionally, the patient had previously experienced temporomandibular joint (TMJ) pain four years ago and had received an appliance at that time, which proved ineffective	Not reported
Mikami *et al*. [78]	2008	36	M	59	Left TMJ	In April 2006, the patient experienced intense pain in the left TMJ region. Despite undergoing physical therapy treatment at a previous medical facility, there was no improvement in the symptoms	Not reported
Naqvi *et al*. [36]	2008	37	M	35	Left TMJ	The patient has been experiencing discomfort and pain in the TMJ area for the past four years. Additionally, he has been suffering from acute severe pain in his left ear, with a potential pustule within the external auditory canal, in conjunction with TMJ discomfort. Although he received treatment for an ear infection, the lesion in the external auditory canal did not resolve	Not reported
Reynolds *et al*. [79]	2008	38	F	52	Left TMJ	The patient presented with longstanding and progressively worsening left TMJ pain. The pain occurred episodically, approximately twice per year, and persisted for 2 weeks during each episode. There were no identifiable precipitating factors. Additionally, the patient experienced regular discomfort when chewing for extended periods on the affected side	Not reported
Covani *et al*. [9]	2009	39	F	74	Right TMJ	Limited mouth opening and severe swelling on the right side of the face. The patient had a three-year history of limited mouth opening and painless swelling	Not reported
Kathju *et al*. [80]	2010	40	F	78	Left TMJ	The patient has reported experiencing intermittent pain in the left TMJ over the course of several years. Additionally, they have noted a gradual increase in swelling on the left cheek over the past year	Hypertension, peripheral vascular disease, history of deep venous thrombosis in both lower extremities, history of bladder cancer, melanoma, transient ischemic attack (TIA), and osteoporosis
Meng *et al*. [47]	2011	41	F	64	Left TMJ	5-year history of chronic pain and swelling	Hypertension, hypercholesterolaemia and a gallbladder stone.
Sklenicka *et al*. [26]	2011	42	F	58	Right TMJ	The patient presents with a 2-month history of progressive swelling of the right TMJ accompanied by trismus and facial pain	Depression, for which she was on paroxetine. Previous surgical history was significant for bunionectomy, lower back surgery, and stapedectomy
Matsumura *et al*. [81]	2012	43	M	46	Right TMJ	Right temporomandibular pain	Mild depression
Nelson *et al*. [31]	2012	44	F	89	Right TMJ	No description	Liposarcoma of the right arm that had been treated with surgical resection 2 years earlier
Srinivasan *et al*. [27]	2012	45	F	51	Right TMJ	The patient presented with a two-month history of left ear pain originating from the TMJ, accompanied by swelling, discomfort while chewing, and mild hearing loss	Not reported
Zweifel *et al*. [48]	2012	46	M	75	Right TMJ	The patient experienced a single episode of TMJ open locking in his right jaw while chewing the previous day. The mandible repositioned spontaneously, but the patient subsequently noticed a slight opening in his bite on the same side. Additionally, he reported localized TMJ pain and swelling	Arterial hypertension, prostate hyperplasia, and malaria in 1994
Lv *et al*. [17]	2013	47	M	62	Right TMJ	Approximately five years ago, the patient experienced unexplained swelling in the anterior region of their right external auditory canal. This swelling was not accompanied by localized pain or limitations in mouth opening. Subsequently, over the past two months, the individual noticed a decline in hearing in their right ear, which was not accompanied by symptoms such as tinnitus, ear pain, or discharge from the ear	History of high blood pressure, high cholesterol, high uric acid, chronic ex- tremity pain, gout or arthritis
Abdelsayed *et al*. [37]	2014	48	M	60	Left TMJ	Limited mouth opening for several years	Not reported
		49	M	75	Right TMJ	The patient has a history of chronic pain in the right jaw accompanied by trismus persisting for 4 years. Over a period of 1 year, the patient experienced pain in the right ear, along with sensations of blockage and thumping tinnitus. Additionally, the patient reported hearing loss and intermittent episodes of positional off-balance sensation. The patient underwent right ear surgery for cholesteatoma	Not reported
		50	F	74	Left TMJ	The patient had been suffering from painful swelling in the left TMJ for five years before seeking medical attention. The pain had intensified significantly in the month leading up to the examination, and the patient had also been experiencing hearing loss in her left ear	Not reported
Laviv *et al*. [54]	2015	51	F	60	Right TMJ	TMJ pain and limited mouth opening for 7 months despite various treatments, affecting daily activities	Generalized degenerative joint disease with symptoms in her back, shoulders, and toes. There was a strong family history of arthritis. No specific diagnosis had been made and routine rheumatoid screens were negative. Her medical history was important for allergy to penicillin, asthma, and thyroid disease. She took levothyroxine, omeprazole, and asthma medications as needed
Kudoh *et al*. [18]	2017	52	M	38	Right TMJ	The patient reported experiencing mild pain in the right chin and tip of the tongue. The onset of mild pain in the right chin occurred 2 months prior to admission, following which the patient received root canal treatment for the right lower second molar	Hyperlipidemia, gout, diabetes, and hyper- tension. These conditions were all well controlled with medication
Fuentes-Martinez *et al*. [62]	2018	53	F	89	Left TMJ	The patient has been experiencing TMJ pain for the past 5 years. She has a history of trauma in that area dating back 40 years; however, the trauma did not necessitate medical attention	Not reported
Kwon *et al*. [82]	2018	54	M	72	Left TMJ	The patient presented with pain and hardening of the area in front of the left ear. He had no significant prior medical conditions and experienced discomfort and crackling sensation when opening his mouth, a condition that had persisted for a couple of years	Not reported
Vellone *et al*. [83]	2018	55	F	64	Right TMJ	Right temporomandibular pain and limitation in mandibular movements	Not reported
De Jong *et al*. [38]	2019	56	M	73	Left TMJ	Twitching of the mouth and the sensation of fullness in the left ear, long-standing dizziness and imbalance	Not reported
Fan *et al*. [39]	2019	57	F	87	Right TMJ	The patient presented with complaints of bilateral ear fullness and a sensation of clogged ears accompanied by a slight decrease in hearing over the past 2 months. Additionally, she reported experiencing chronic facial pain that began 2 years ago, which has progressively worsened over the last 4 months. The pain was exacerbated during jaw movement. Furthermore, she noted the presence of pain on the right side of her throat, anosmia, and a loss of ability to taste food over the past 2 months	Chronic kidney disease, congestive heart failure, hypertension, lung nodules, and COPD
Sha *et al*. [84]	2019	58	F	57	Right TMJ	Right TMJ pain for more than 5 years.	Not reported
Abou-Foul *et al*. [53]	2020	59	M	56	Left TMJ	Progressive left sided TMJ discomfort, swelling and trismus	Not reported
Choi *et al*. [19]	2020	60	F	61	Bilateral TMJ	The patient presented with initial complaints of bilateral TMJ pain and a history of decreased mouth opening spanning several years	Not reported
		61	F	66	Bilateral TMJ	The patient initially reported experiencing limited mouth opening and persistent joint pain subsequent to a motor vehicle accident that occurred 8 months ago	Hypothyroidism
		62	M	72	Right TMJ	The patient presented with acute pain in the right TMJ, swelling in front of the ear, and restricted mouth opening. He had a 20-year history of right TMJ disease, which was managed through non-invasive methods	Gout, type II diabetes, and essential tremors
Gomez Serrano *et al*. [40]	2020	63	M	73	Left TMJ	Left-sided hearing loss	Not reported
Hotokezaka *et al*. [10]	2020	64	F	59	Left TMJ	The patient, with a 4-year history of left cheek swelling, pain and trismus, was referred due to acute exacerbation of left cheek pain and trismus	Not reported
Houghton *et al*. [85]	2020	65	F	55	Right TMJ	The patient presents with a painless right-sided pre-auricular mass that has been progressively increasing in size over the past 2 years	Not reported
Loro *et al*. [86]	2020	66	F	40	Bilateral TMJ	The patient presented with swelling in the left preauricular region. She had a significant history of TMJ pain and restricted jaw movements. Fourteen years prior, she underwent bilateral discectomy and synovectomy. Subsequently, three years ago, she underwent arthroplasty and synovectomy in the right TMJ due to severe pain and substantial reduction in TMJ function	Asthma, hypertension, and psoriatic arthritis with involvement of both TMJs diagnosed over 20 years ago
Tang *et al*. [41]	2021	67	F	46	Right TMJ	The patient has been experiencing persistent pain and discomfort in the right temporal region for a duration exceeding three months	Diabetes mellitus
		68	M	52	Left TMJ	The patient has been experiencing a mass in the left TMJ for the past 6 years	Not reported
Bschorer *et al*. [8]	2022	69	F	53	Left TMJ	The patient presented with progressive pain in the left TMJ and noted a progressive asymmetry of the face attributed to swelling in the left preauricular region. She reported that these symptoms had slowly developed over the past decade	Not reported
Dang *et al*. [49]	2022	70	M	65	Left TMJ	The patient has been experiencing a progressively worsening left posterior open bite and mild, dull, constant left pre-auricular tenderness for the past 4–5 months	Hypertension and ulcerative colitis
Murahashi *et al*. [87]	2022	71	M	61	Right TMJ	The patient has been experiencing persistent preauricular pain in the right cheek and restricted mouth opening for more than 6 months	Hypertension and type 2 diabetes mellitus
Takeda *et al*. [63]	2022	72	F	83	Right TMJ	First noticed the pain 3 years earlier	Not reported
Terauchi *et al*. [55]	2022	73	M	47	Right TMJ	Persistent pain in the right TMJ and trismus	Not reported
Bukawa *et al*. [21]	2023	74	F	73	Left TMJ	Acute swelling at the closure site of the left ear	Hypertension, osteoporosis, and hyperlipidemia, and she has been receiving anti-RANKL preparations for 2 years, she also took an angiotensin II receptor blocker, pravastatin sodium

*Legend: M: Male; F: Female; MRI: Magnetic Resonance; COPD: Chronic obstructive pulmonary disease; RANKL: receptor activator of nuclear factor kappa beta ligand.*

### 4.2 Signs and symptoms

Out of the 74 cases, there were only 12 patients [16, 21, 22, 23, 24, 25, 26, 27, 28] reporting sudden symptom, such as pain or dysfunction for less than 3 months. Three of those cases [29, 30, 31] did not have a description of the clinical history of the chief complaint, while the remaining 59 patients have been experiencing chronic signs and symptoms for several years. At the time of diagnosis, 83.7% of patients (62 out of 74) reported experiencing pain. Additionally, 71.6% (53 out of 74) exhibited both objective (a decrease in interincisal distance to less than 35 mm) and subjective mouth-opening limitation. Swelling was the third most common clinical sign, observed in 40.54% of patients, with 30 out of 74 individuals experiencing this symptom. Furthermore, 14 patients [16, 17, 28, 32, 33, 34, 35, 36, 37, 38, 39, 40, 41, 42] were referred for hearing loss, which was confirmed in all cases by instrumental analysis. Finally, a total of 11 patients [23, 35, 43, 44, 45, 46, 47, 48, 49, 50], which accounts for 14.86% of the group, reported experiencing changes in their dental occlusion. All the signs and symptoms summarized could be found in Table 2 (Ref. [20, 21, 22, 23, 24, 25, 26, 27, 28, 29, 30, 31, 32, 33, 34, 35, 36, 37, 38, 39, 40, 41, 42, 43, 44, 45, 46, 47, 48, 49, 50, 51, 52, 53, 54, 55, 56, 57, 58, 59, 60, 61, 62, 63, 64, 65, 66, 67, 68, 69, 70, 71, 72, 73, 74, 75, 76, 77, 78, 79, 80, 81, 82, 83, 84, 85, 86, 87]).

**Table 2.** Shows the patients referring pain, occlusal dental changes, jaw dysfunction (limited mouth opening) and hearing loss.

Study first Authors	Year	N of patients	Occlusal changes	Pain	Jaw disfunction	Hearing loss
Good *et al*. [20]	1982	1	0	1	1	0
Zemplenyi *et al*. [29]	1985	2	0	1	1	0
Gross *et al*. [30]	1987	3	0	1	1	0
Hutton *et al*. [22]	1987	4	0	1	1	0
		5	0	1	1	0
		6	0	1	1	0
Kamatani *et al*. [43]	1987	7	1	0	1	0
Mogi *et al*. [61]	1987	8	0	1	1	0
Lambert *et al*. [32]	1990	9	0	0	1	1
Dijkgraaf *et al*. [44]	1992	10	1	1	1	0
Magno *et al*. [33]	1992	11	0	1	0	1
Chuong *et al*. [46]	1995	12	1	1	1	0
Pynn *et al*. [45]	1995	13	1	0	0	0
Allias-Montmayeur *et al*. [23]	1997	14	1	1	1	0
		15	1	1	1	0
Kurihara *et al*. [51]	1997	16	0	1	0	0
Onodera *et al*. [69]	1997	17	0	0	1	0
Vargas *et al*. [70]	1997	18	0	1	1	0
Jordan *et al*. [16]	1998	19	0	0	0	1
Strobl *et al*. [71]	1998	20	0	1	1	0
Goudot *et al*. [72]	1999	21	0	1	1	0
Grant *et al*. [28]	1999	22	0	1	1	1
Nakagawa *et al*. [73]	1999	23	0	1	1	0
		24	0	1	0	0
Pynn *et al*. [50]	1998	25	1	0	0	0
Aoyama *et al*. [74]	2000	26	0	1	1	0
Li-Yu *et al*. [34]	2000	27	0	1	1	1
Eriksson *et al*. [75]	2001	28	0	1	1	0
Olin *et al*. [24]	2001	29	0	0	0	0
Osano *et al*. [76]	2003	30	0	1	1	0
Marsot-Dupuch *et al*. [35]	2004	31	1	1	1	1
		32	0	1	1	1
Meul *et al*. [77]	2005	33	0	1	1	0
Smolka *et al*. [25]	2005	34	0	1	1	0
Nicholas *et al*. [52]	2007	35	0	1	0	0
Mikami *et al*. [78]	2008	36	0	1	0	0
Naqvi *et al*. [36]	2008	37	0	1	0	1
Reynolds *et al*. [79]	2008	38	0	1	1	0
Covani *et al*. [9]	2009	39	0	1	1	0
Kathju *et al*. [80]	2010	40	0	1	1	0
Meng *et al*. [47]	2011	41	1	1	1	0
Sklenicka *et al*. [26]	2011	42	0	1	1	0
Matsumura *et al*. [81]	2012	43	0	1	1	0
Nelson *et al*. [31]	2012	44	0	1	0	0
Srinivasan *et al*. [27]	2012	45	0	0	1	0
Zweifel *et al*. [48]	2012	46	1	1	0	0
Lv *et al*. [17]	2013	47	0	0	0	1
Abdelsayed *et al*. [37]	2014	48	0	1	1	0
		49	0	1	1	1
		50	0	1	0	0
Laviv *et al*. [54]	2015	51	0	1	1	0
Kudoh *et al*. [18]	2017	52	0	1	1	0
Fuentes-Martinez *et al*. [62]	2018	53	0	1	0	0
Kwon *et al*. [82]	2018	54	0	1	1	0
Vellone *et al*. [83]	2018	55	0	1	1	0
De Jong *et al*. [38]	2019	56	0	1	0	1
Fan *et al*. [39]	2019	57	0	1	1	1
Sha *et al*. [84]	2019	58	0	1	0	0
Abou-Foul *et al*. [53]	2020	59	0	1	1	0
Choi *et al*. [19]	2020	60	0	1	1	0
		61	0	1	1	0
		62	0	1	1	0
Gomez Serrano *et al*. [40]	2020	63	0	0	0	1
Hotokezaka *et al*. [10]	2020	64	0	1	1	0
Houghton *et al*. [85]	2020	65	0	0	0	0
Loro *et al*. [86]	2020	66	0	1	1	0
Tang *et al*. [41]	2021	67	0	1	1	0
		68	0	1	0	1
Bschorer *et al*. [8]	2022	69	0	1	0	0
Dang *et al*. [49]	2022	70	1	1	1	0
Murahashi *et al*. [87]	2022	71	0	1	1	0
Takeda *et al*. [63]	2022	72	0	1	1	0
Terauchi *et al*. [55]	2022	73	0	1	1	0
Bukawa *et al*. [21]	2023	74	0	0	1	0

*Legend: 0: not present; 1: present.*

### 4.3 Blood exam

Blood tests were conducted on 28 patients as listed in Table 3 (Ref. [20, 21, 22, 23, 24, 25, 26, 27, 28, 29, 30, 31, 32, 33, 34, 35, 36, 37, 38, 39, 40, 41, 42, 43, 44, 45, 46, 47, 48, 49, 50, 51, 52, 53, 54, 55, 56, 57, 58, 59, 60, 61, 62, 63, 64, 65, 66, 67, 68, 69, 70, 71, 72, 73, 74, 75, 76, 77, 78, 79, 80, 81, 82, 83, 84, 85, 86, 87]). The majority of patients had normal biochemistry results, except for a few exceptions:

- Kurihara *et al*. [51] observed a very slight elevation of hyperuricemia;

- Grant *et al*. [28] highlighted elevated levels of serum calcium; ionized calcium; parathyroid hormone; and a low phosphate level;

- Nicholas *et al*. [52] demonstrated an elevated intact parathyroid hormone level and hypocalcemia;

- Meng *et al*. [47] reported a slightly higher phosphate and cholesterol;

- Abou-Foul *et al*. [53] identified a slightly elevated parathyroid hormone.

**Table 3.** Summary of blood examination.

Study first Author	Year	N of patients	Blood exams
Good *et al*. [20]	1982	1	Normal biochemistry
Zemplenyi *et al*. [29]	1985	2	Normal biochemistry
Gross *et al*. [30]	1987	3	Not reported
Hutton *et al*. [22]	1987	4	Not reported
		5	Not reported
		6	Not reported
Kamatani *et al*. [43]	1987	7	Normal biochemistry
Mogi *et al*. [61]	1987	8	Normal biochemistry
Lambert *et al*. [32]	1990	9	Normal biochemistry
Dijkgraaf *et al*. [44]	1992	10	Not reported
Magno *et al*. [33]	1992	11	Not reported
Chuong *et al*. [46]	1995	12	Not reported
Pynn *et al*. [45]	1995	13	Not reported
Allias-Montmayeur *et al*. [23]	1997	14	Not reported
		15	Not reported
Kurihara *et al*. [51]	1997	16	Slight hyperuricemia (8.1 mg/dL)
Onodera *et al*. [69]	1997	17	Not reported
Vargas *et al*. [70]	1997	18	Not reported
Jordan *et al*. [16]	1998	19	Not reported
Strobl *et al*. [71]	1998	20	Normal biochemistry
Goudot *et al*. [72]	1999	21	Normal biochemistry
Grant *et al*. [28]	1999	22	Normal biochemistry except for elevated levels of serum calcium (11.9 mg/dL; normal range 8.9–10.2 mg/dL); ionized calcium (1.38 mmol/L; normal range 1.16–1.27 mmol/L); parathyroid hormone (122 pg/mL; normal range 21–69 pg/mL); and a low phosphate level (2.8 mg/dL; normal range 3–4.5 mg/dL)
Nakagawa *et al*. [73]	1999	23	Normal biochemistry
		24	Not reported
Pynn *et al*. [50]	1998	25	Not reported
Aoyama *et al*. [74]	2000	26	Not reported
Li-Yu *et al*. [34]	2000	27	Normal biochemistry except for an elevated erythrocyte sedimentation rate of 66 mm/hr
Eriksson *et al*. [75]	2001	28	Not reported
Olin *et al*. [24]	2001	29	Not reported
Osano *et al.* [76]	2003	30	Normal biochemistry
Marsot-Dupuch *et al*. [35]	2004	31	Not reported
		32	Not reported
Meul *et al*. [77]	2005	33	Normal biochemistry
Smolka *et al*. [25]	2005	34	Normal biochemistry
Nicholas *et al*. [52]	2007	35	Elevated intact parathyroid hormone level and hypocalcemia with no other metabolic abnormalities.
Mikami *et al*. [78]	2008	36	Not reported
Naqvi *et al*. [36]	2008	37	Not reported
Reynolds *et al.* [79]	2008	38	Normal biochemistry except for slight elevation in erythrocyte sedimentation rate to 22 (upper limit of normal 21)
Covani *et al*. [9]	2009	39	Normal biochemistry
Kathju *et al*. [80]	2010	40	Not reported
Meng *et al*. [47]	2011	41	Normal biochemistry except for lightly higher phosphate, 1.49 mmol/L (normal 0.81–1.46) and cholesterol 5.89 mmol/L (normal 3.10– 5.70)
Sklenicka *et al*. [26]	2011	42	Not reported
Matsumura *et al*. [81]	2012	43	Normal biochemistry, except for a positive hepatitis virus Type B infection
Nelson *et al*. [31]	2012	44	Not reported
Srinivasan *et al*. [27]	2012	45	Not reported
Zweifel *et al*. [48]	2012	46	Normal biochemistry
Lv *et al*. [17]	2013	47	Normal biochemistry
Abdelsayed *et al*. [37]	2014	48	Not reported
		49	Not reported
		50	Not reported
Laviv *et al*. [54]	2015	51	Normal biochemistry
Kudoh *et al*. [18]	2017	52	Not reported
Fuentes-Martinez *et al*. [62]	2018	53	Not reported
Kwon *et al*. [82]	2018	54	Not reported
Vellone *et al*. [83]	2018	55	Normal biochemistry
De Jong *et al*. [38]	2019	56	Not reported
Fan *et al*. [39]	2019	57	Not reported
Sha *et al*. [84]	2019	58	Normal biochemistry
Abou-Foul *et al*. [53]	2020	59	Slightly elevated parathyroid hormone but otherwise normal biochemistry
Choi *et al*. [19]	2020	60	Not reported
		61	Not reported
		62	Not reported
Gomez Serrano *et al*. [40]	2020	63	Not reported
Hotokezaka *et al*. [10]	2020	64	Not reported
Houghton *et al*. [85]	2020	65	Normal biochemistry
Loro *et al*. [86]	2020	66	Normal biochemistry
Tang *et al*. [41]	2021	67	Not reported
		68	Not reported
Bschorer *et al*. [8]	2022	69	Not reported
Dang *et al*. [49]	2022	70	Normal biochemistry
Murahashi *et al*. [87]	2022	71	Normal biochemistry
Takeda *et al*. [63]	2022	72	Normal biochemistry
Terauchi *et al*. [55]	2022	73	Normal biochemistry
Bukawa *et al*. [21]	2023	74	Not reported

### 4.4 Radiological examination

In Table 4, the radiological examinations performed for the 74 patients were reported to achieve a diagnosis. 63 out of 74 (85.13%) had been studied with CT. Out of the total number of patients, 41 exhibited condylar degeneration. However, in 33 cases, there were no radiological signs of invasion of nearby tissues. Twenty-two patients experienced temporal bone resorption, with an invasion of the skull base by CPPD and affection of the meninges in 2 cases. 8 patients experienced a local invasion in the ear canal. Finally, in 2 cases, the Magnetic Resonance (MRI) revealed an involvement of the parotid gland by the neoformation.

In 9 cases [18, 21, 22, 23, 30, 44, 52] CPPD lesions were also identified in various joints, with knee and wrist being the most commonly involved.

**Table 4.** Radiological examination performed to study CPPD.

Radiological Examination	N of Patients
Only CT	30
Only MRI	4
Both CT and MRI	29
Ultrasound	0
RX (orthopantomography)	7
Both CT and RX	4

*Legend: CT: computerized tomography; MRI: Magnetic Resonance; RX: Radiography.*

### 4.5 Diagnosis of CPPD

Out of the total 74 cases of CPPD, only three Authors [18, 22, 55] were able to identify it through radiological examination. In 21 cases, joint aspiration was performed to complete the diagnostic process. In 50 patients, the diagnostic process itself served as the treatment solution with complete removal of the lesion.

### 4.6 Treatment, relapse and follow-up

Table 5 reports the various methods used by different Authors to treat CPPD. The surgical approach is the most common, with varying degrees of invasiveness. The three papers that managed patients solely with drugs utilized different solutions.

**Table 5.** Various methods used to treat CPPD.

Type of Treatment	N of Patients
Only Follow-up	12
Medications	3
Surgical removal of the lesion	26
Arthroplasty and removal of the lesion	13
Condylectomy, removal of the lesion and joint reconstruction	16
Massive demolition (condyle, glenoid fossa, zygomatic arch, muscles…) and joint reconstruction	4

- In the study by Good *et al*. [20] Indomethacin, administered at a dosage of 25 mg four times daily, was found to provide relief from pain within a few hours.

- Choi *et al*. [19] treated one of the three patients with colchicine without benefit, but reported an improvement with non-steroidal anti-inflammatory drugs (NSAIDs) during acute exacerbations. The second case in their case series was treated with a steroid injection in the TMJ. In both cases the pharmacological treatment was not detailed.

Only one relapse of CPPD was reported, in the paper of Dijkgraaf *et al*. [44], whose patient underwent arthroplasty and surgical removal of the lesion. However, after one year, he received further treatment which involved the insertion of temporalis fascia. This resulted in the resolution of symptoms and an improvement in function. Patients who only received follow-up or pharmacological treatment did not experience any changes in the lesion or symptoms during the follow-up period.

The follow-up period averaged 13.45 ± 23 months.

## 5. Discussion

A systematic assessment of the literature on CPPD of the TMJ over the past decades allowed the identification of 61 new cases since the last comprehensive review by Pynn *et al*. [45], thus reaching a total of 75 cases, included the case reported in this manuscript. It is common for studies to focus on individual cases, with only a small number of publications describing multiple cases (only 6 out of 74). The current review assessed systematically all available publications from 1980 to date, providing a standpoint for describing the disease’s epidemiological characteristics.

Pseudogout, also known as Calcium pyrophosphate dihydrate deposition disease (CPPD), is a metabolic condition characterized by the accumulation of calcium pyrophosphate dihydrate crystals in the synovial fluid. This accumulation leads to the calcification of articular cartilage and can cause acute arthritis in a small subset of patients [56]. Various etiologies were proposed [57]: Hereditary, Sporadic (idiopathic), Associated with metabolic disease (Hyperparathyroidism, reaching, Hypophosphatasia, Rheumatoid arthritis, Hemochromatosis, Hypomagnesemia and Renal failure), Associated with trauma or surgery.

CPPD often develops in joints containing fibrocartilage such as the knee, wrist, hip, shoulder and elbow. While involvement in the TMJ is rare, it is common to find lesions larger than 1 cm. In some cases, the crystals cause complete destruction of the joint space and invade surrounding structures: masticator space, parotid space and skull base [58].

### 5.1 Population’s characteristics

CPPD is usually of interest to middle-aged or older patients [45], as confirmed by their average age at the time of diagnosis reported in this review, which is around 60 years. Additionally, females are affected 1.6 times more frequently by this condition than males, and this trend has remained consistent across recent and past studies. The vast majority of cases (around 95%) showed a unilateral TMJ involvement as reported by Tamimi *et al*. [58].

### 5.2 Signs and symptoms and blood test evaluation

The most common symptoms of this condition include pain (83.7% of patients), reduced mouth opening (71.6%), and swelling (40.5% of patients). Additionally, there are less common symptoms such as hearing loss (18.9%) and changes in dental occlusion (14.8%). These are common clinical manifestations of various temporomandibular joint disorders, such as chondromatosis of the TMJ [59], so that differential diagnosis may be a concern that explains a potential late diagnosis [60].

There are no frequently altered blood tests; the few papers [28, 47, 51, 52, 53] that had studied the patient by a hematological point of view reported only minimal variations that are not relevant for diagnostic purposes.

### 5.3 Radiological examination

Imaging techniques such as MRI and CT can be useful in identifying joint changes. These techniques can also reveal the presence of cloud-like synovial calcification in cases of early/mild CPPD. In late/severe stages, a chunky, diffusely calcified mass with a ground-glass appearance may be detected [58]. MRI can be used to accurately detect the presence of calcification in early CPPD, while in severe stages with invasion of muscles [43, 61], parotid gland [29] and even brain structure [16], aiding in identifying soft tissue involvement and planning surgical treatment.

Williams *et al*. [57] had reported that TMJ is more commonly involved than previously reported in patients affected by peripheral calcific disease, with a higher ratio females:males. This was confirmed by 9 Authors [18, 20, 22, 23, 30, 44, 54] who showed the presence of a synchronous involvement of different major joints, in particular the knee and the wrist.

### 5.4 Treatment, relapse and follow-up

The presence of multiple crystals of calcium pyrophosphate dihydrate in the joint space can disrupt its function and may require surgical removal. This review found that out of 74 cases, 59 were treated surgically using various methods such as removal of the lesion, arthroplasty with lesion removal, condylectomy with joint replacement, and complete demolition with joint replacement. It is difficult to determine the superiority of any method over another due to the heterogeneity of the study samples in terms of type, duration, and size of the lesion. Additionally, early stage lesions may just be monitored during follow-up or treated with medication based on symptoms or joint lavage, which showed a very good effectiveness for several TMJ internal derangements [18, 19, 20, 21, 22, 35, 37, 39, 62, 63, 64, 65].

### 5.5 Limitations

The included source studies exhibited heterogeneity in terms of diagnosis, treatment and follow-up, making it more suitable to conduct separate meta-analyses for the diagnosis/prevalence of CPPD in the TMJ and the different outcomes of treatment. However, the limited number of studies in this systematic review precludes their division into subgroups. The search was conducted in English, potentially resulting in the omission of records unindexed with English keywords. Due to the scarcity of source material, studies with uncertain and high risk of bias were included in the synthesis.

### 5.6 Strengths

The strengths of this systematic review encompass the meticulous definition of eligibility criteria, the comprehensive nature of the search, and strict adherence to PRISMA guidelines.

The case report outlined in this paper has many similarities to the other cases described in the literature. Late diagnosis was a common theme, as suggested by previous research, and it must be remarked that dentists unfortunately play a negative role due to the focus on oral appliance treatment and/or occlusal approaches as a one-fits-all approach to all TMJ disorders [12, 66]. Such a concern was also pointed out in previous papers on late-stage surgical demanding lesions [67]. Imaging techniques were used to have an accurate depiction of the joint status and plan for surgery, in line with current standards [68]. Open surgery was chosen as the best option to remove the sizable lesion detected with CT. The postoperative recovery was smooth, indicating a positive prognosis and successful treatment outcome, consistent with other instances of CPPD of the TMJ.

As a general remark, data reported in this review could help researchers pinpoint areas for improvement in future studies. To enhance knowledge on CPPD of the TMJ, there must be better quality literature on the disease. Many case reports lacked information on the disease stages as well as detailed surgical technique descriptions. Future research should aim to furnish comprehensive details regarding the patient’s medical history, encompassing anamnesis and the duration of symptoms. This approach is essential for identifying potential risk factors associated with the onset and progression of the disease.

## 6. Conclusions

In conclusion, CPPD is a rare and locally invasive benign disease that seldom affects the TMJ. A case report of a patient exhibiting classic CPPD symptoms has been presented, along with a comprehensive review of related literature from the past few decades. The review included 74 cases of CPPD in the TMJ, with a ratio of 1.6 females to every male and an average age of around 60 years. Bilateral localization was observed only in four cases. Although all cases showed varying degrees of hard and soft tissue invasion, thus making it not possible to generalize findings, there is a lack of information regarding the relationship between a possible extra-articular extension and time. In any case, late diagnosis is a common concern due to the often-unspecific symptoms. While surgery has the preferred treatment option for most of the authors and is mandatory for late stage lesions, a conservative approach may be considered in the early stages. The recurrence rate is extremely low, with only one case reported in the literature.

This review has demonstrated its efficacy in establishing the correct pathway for the diagnosis and management of suspected TMJ CPPD cases.

Further research is needed to establish a disease staging system based on the involvement of both articular and extra-articular tissues. This will, in turn, facilitate the standardization of TMJ CPPD treatment according to the localized invasiveness of the lesion.

## Supplementary Material

Supplementary material associated with this article can be found, in the online version, at https://files.jofph.com/ files/article/1899699082825220096/attachment/ Supplementary%20material.docx.


